# Supplementary material for: Assessing and refining a metabolite-based distress score for use in different populations within three US cohorts
Source: medRxiv. 2026 Sep 13:2026.09.10.26362734. Preprint. [Version 1] doi: 10.64898/2026.09.10.26362734 (PMC13622792; doi:10.64898/2026.09.10.26362734)
Supplement: Supplementary file 1 [file NIHPP2026.09.10.26362734v1-supplement-1.pdf]

Supplemental Table 1. Covariate assessment in each participating cohort

Covariates	MESA	WHI	NHS
Age	Age was derived from date of birth collected at the baseline clinic visit and analyzed as a continuous variable in years.	Age was self-reported at baseline and analyzed as a continuous variable in years.	Age was derived from date of birth collected at enrollment and date of blood collection, analyzed as a continuous variable in years.
Sex	Gender/sex was recorded at the baseline exam from participant survey and treated as a demographic covariate in analyses.	NA (Only women were considered eligible for enrollment)	NA (Only women were considered eligible for enrollment)
Study site	Study site was defined by the participant's MESA field center (Baltimore, Chicago, Forsyth County, Los Angeles, New York, or St. Paul) at enrollment/baseline examination.	Annual household income was self-reported aat baseline, and was recorded as "<$20,000", "$20,000- 49,999", "$50,000- 99,999", and "≥$100,000"	NA (Not a multi-site design)
Education	Educational attainment was collected by questionnaire as part of baseline survey obtained during the clinic exam and was recoded as "high school of less", "some college", "college graduate", and "graduate school"	Not available	Not available

medRxiv preprint doi: https://doi.org/10.64898/2026.09.10.26362734; this version posted September 13, 2026. The copyright holder for this preprint (which was not certified by peer review) is the author/funder, who has granted medRxiv a license to display the preprint in perpetuity.
It is made available under a CC-BY 4.0 International license .

Income	Annual household income was self-reported via baseline questionnaire as part of socioeconomic information collected at the baseline exam, and was recoded as "<$20,000", "$20,000- 49,999", "$50,000- 99,999", and "≥$100,000"	Annual household income was self-reported aat baseline, and was recorded as "<$20,000", "$20,000- 49,999", "$50,000- 99,999", and "≥$100,000"	Because no individual household income measure was available, we obtained the median household income of the participants' census tract (derived from geocoded addresses) based on the 1990 census. The average of three available measures prior to the blood collection (1986, 1988, 1990) was analyzed as a continuous variable (unit: dollar).
Employment status	Employment status was collected by questionnaire as part of baseline survey, and was recoded as "homemaker", "employed", "unemployed", and "retired".	Not available	Not available (Occupational cohort with minimal variation at baseline)
Body mass index (BMI)	Weight and standing height were measured by trained staff using standardized anthropometry procedures at the clinic exam; BMI was computed as weight (kg) / height (m^2).	Height and weight were measured by trained staff at baseline and used to calculate BMI (kg/m2).	BMI at blood collection was derived using height reported at study entry and weight reported at blood collection (kg/m2).
Smoking status	Cigarette smoking status was obtained by questionnaire and classified as never/former/current based on standard definitions.	Information on smoking status was self-reported on the lifestyle and medical history questionnaires at baseline. Participants were classified as current smokers vs not current smokers.	Smoking status was self-reported in a biennial questionnaire closest to the blood collection (1988) and we classified participants as current smokers vs not current smokers.

medRxiv preprint doi: https://doi.org/10.64898/2026.09.10.26362734; this version posted September 13, 2026. The copyright holder for this preprint (which was not certified by peer review) is the author/funder, who has granted medRxiv a license to display the preprint in perpetuity.
It is made available under a CC-BY 4.0 International license .

Physical activity	Physical activity was assessed using the MESA Typical Week Physical Activity Survey, which captured frequency and duration of activities during a typical week in the past month, summarized as MET-hrs/week and analyzed as a continuous variable.	Physical activity was assessed using a brief recreational physical activity inventory and calculated as MET-hrs/week and analyzed as a continuous variable.	Physical activity was self-reported in biennial questionnaires closest to the blood collection (1986, 1988) and the average MET-hrs/week for total activity was analyzed as a continuous variable.
Diet quality	Usual dietary intake over the prior year was assessed using a 120-item food frequency questionnaire modified from a validated instrument. Diet quality was assessed by the Alternate Healthy Eating Index scores derived from the FFQ according to the AHEI-2010 scoring algorithm and analyzed as a continuous variable	Dietary intake was self-reported at baseline, assessed with a Food Frequency Questionnaire (FFQ), from which an overall Healthy Eating Index (HEI-2005) score was derived. The score was analyzed as a continuous variable.	Dietary intake was assessed using the Food Frequency Questionnaire at three biennial surveys prior to the blood collection (1984, 1986, 1990) and the average derived Alternate Healthy Eating Index score (according to the AHEI-2010 scoring algorithm) was analyzed as a continuous variable.
Systolic blood pressure (SBP)	Seated blood pressure was measured at the baseline clinic exam using an automated Dinamap device; multiple readings were obtained, and the mean of the 2nd and 3rd measurements for systolic blood pressure was used in the analysis as a continuous variable	At the baseline study visit, seated blood pressure was measured by certified staff in the right arm with a mercury sphygmomanometer. The average of two blood pressure readings was used for analysis as a continuous variable.	Systolic blood pressure was self-reported in categories as part of the 1988 questionnaire, with the following levels: <115mmHg , 115-124mmHg, 125-134mmHg, 135-144mmHg, 145-154mmHg, 155-164mmHg, 165-174mmHg, ≥ 175mmHg. We analyzed this measure as an ordinal variable.

medRxiv preprint doi: https://doi.org/10.64898/2026.09.10.26362734; this version posted September 13, 2026. The copyright holder for this preprint (which was not certified by peer review) is the author/funder, who has granted medRxiv a license to display the preprint in perpetuity.
It is made available under a CC-BY 4.0 International license .

HDL cholesterol	HDL-C was measured from fasting blood samples collected at baseline and analyzed at a central laboratory in University of Vermont using standardized laboratory procedures. HDL-C was analyzed as a continuous variable.	HDL cholesterol was obtained from fasting blood samples obtained at the baseline study visit and analyzed as a continuous variable (mg/dL).	Not available
Total cholesterol	Total cholesterol was measured from fasting blood samples collected at baseline and analyzed at a central laboratory in University of Vermont using standardized laboratory procedures. Total cholesterol was analyzed as a continuous variable.	Total cholesterol was obtained from fasting blood samples obtained at the baseline study visit and analyzed as a continuous variable (mg/dL).	Not analyzed (only reported as a categorical variable and missing in over 30% of the study sample)
Diabetes status	Diabetes status at baseline was defined as fasting glucose ≥126 mg/dL and/or use of diabetes medication and/or self-reported physician diagnosis, and was analyzed as a binary variable.	History of diabetes was assessed at the baseline interview and analyzed as a binary variable.	Not analyzed (<10% participants reported diabetes history, and no Black participant with depression endorsed having diabetes history)
Antihypertensive medications	Medication use was ascertained via a baseline medication inventory: participants were asked to bring containers for medications used in the prior ~2 weeks, and staff transcribed medication name/strength/frequency while confirming actual use with the participant; antihypertensives were identified from this inventory.	Use of antihypertensive medication was assessed at the baseline interview and analyzed as a binary variable.	Not analyzed (no Black participant with depression endorsed use of antihypertensive medications)

medRxiv preprint doi: https://doi.org/10.64898/2026.09.10.26362734; this version posted September 13, 2026. The copyright holder for this preprint (which was not certified by peer review) is the author/funder, who has granted medRxiv a license to display the preprint in perpetuity.
It is made available under a CC-BY 4.0 International license .

Lipid-lowering medications	Lipid-lowering therapy use was ascertained using the same baseline medication inventory procedure, and lipid-lowering agents were identified from recorded medication data.	Use of lipid-lowering medication was assessed at the baseline interview and analyzed as a binary variable.	Not analyzed (<10% participants reported use of lipid lowering medication, and no Black participant with depression endorsed use)
Menopausal status	Self-reported	All postmenopausal	Menopausal status and use of post-menopausal hormone therapy (PMH) were self-reported at blood collection. Each participant's status was classified as premenopausal, postmenopausal but not on PMH, postmenopausal and on PMH, or unknown. This information was included in the analyses as a categorical variable.

medRxiv preprint doi: https://doi.org/10.64898/2026.09.10.26362734; this version posted September 13, 2026. The copyright holder for this preprint (which was not certified by peer review) is the author/funder, who has granted medRxiv a license to display the preprint in perpetuity.
It is made available under a CC-BY 4.0 International license .

Supplemental Table 2. List of 20 metabolites previously associated with depression in White women.

Metabolites	HMDB ID	Weights
3-methylxanthine	HMDB01886	0.329765903
biliverdin	HMDB01008	-0.423583685
cotinine	HMDB01046	0.366219583
GABA	HMDB00112	-0.152260834
N4-acetylcytidine	HMDB05923	-0.11936796
pseudouridine	HMDB00767	0.049966016
serotonin	HMDB00259	-0.586268696
threonine	HMDB00167	-0.436917435
creatinine	HMDB00562	-0.358052338
glutamine	HMDB00641	0.372924654
N2,N2-dimethylguanosine	HMDB04824	0.574745872
thiamine	HMDB00235	0.046196797
tryptophan	HMDB00929	-0.246402337
C16:0 Ceramide (d18:1)	HMDB04949	0.129344695
C18:0 LPE	HMDB11130	0.1123545
C34:3 PC	HMDB08006	-0.400454449
C36:5 PC plasmalogen-B	HMDB11220	-0.1138951
C38:3 PC	HMDB08047	0.307754297
hippurate	HMDB00714	-0.425864438
arachidonate	HMDB01043	-0.329996729

medRxiv preprint doi: https://doi.org/10.64898/2026.09.10.26362734; this version posted September 13, 2026. The copyright holder for this preprint (which was not certified by peer review) is the author/funder, who has granted medRxiv a license to display the preprint in perpetuity.
It is made available under a CC-BY 4.0 International license .

Supplemental Table 3. Sample characteristics in White and Black women from WHI and NHS

	WHI		NHS	
	White	Black	White	Black
N	1,107	149	1856	214
CESD	2.3 (2.5)	2.9 (2.6)	-	-
CESD≥16, %	15.3	20.8	-	-
MHI-5	-	-	78.0 (13.5)	77.8 (14.0)
MHI-5 ≤52, %	-	-	5.6	7.3
Antidepressant use, %	7.1	4.7	6.4	2.8
History of depressed mood, %	10.8	15.4	-	-
Depression diagnosis, %	-	-	5.5	3.3
Depression status, %	24.1	28.9	12.4	10.3
**Sociodemographic factors**				
Age, yrs	67.2 (6.8)	64.2 (6.8)	55.6 (6.9)	56.1 (6.4)
Household income, %				
<$20,000	28.9	55.0	-	-
$20,000-$49,999	52.1	36.2	-	-
$50,000-$99,999	15.8	7.4	-	-
≥$100,000	3.2	1.3	-	-
Census tract-based median household income, $	-	-	46766 (17087)	39580 (16563)
**Medical factors**				
Menopausal status and postmenopausal hormone (PMH) use, %				
Premenopausal	-	-	25.6	15.9
Postmenopausal but not on PMH	-	-	30.7	50.9
Postmenopausal, on PMH	-	-	31.2	22.9
Unknown status	-	-	12.4	10.3
Systolic blood pressure	132.8 (18.3)	138.3 (20.6)	-	-
Self-reported systolic blood pressure categories				
< 115 mmHg	-	-	24.8	18.6
115 - 124 mmHg	-	-	26.8	22.6
125 - 134 mmHg	-	-	30.6	30.7

medRxiv preprint doi: https://doi.org/10.64898/2026.09.10.26362734; this version posted September 13, 2026. The copyright holder for this preprint (which was not certified by peer review) is the author/funder, who has granted medRxiv a license to display the preprint in perpetuity.
It is made available under a CC-BY 4.0 International license .

135 - 144 mmHg	-	-	11.3	18.1
145 - 154 mmHg	-	-	5.1	8.0
155 - 164 mmHg	-	-	1.0	1.5
165 - 174 mmHg	-	-	0.2	0.5
≥ 175 mmHg	-	-	0.2	0
HDL cholesterol, mg/dL	52.3 (13.2)	48.9 (12.4)	-	-
Total cholesterol, mg/dL	235.5 (48.4)	237.3 (40.0)	-	-
Diabetes status, %[1]	22.4	13.5	3.0	6.5
Antihypertensive medications, %[1]	46.8	27.6	13.0	20.6
Lipid-lowering medications, %[1]	15.4	15.6	2.2	2.8
**Lifestyle factors**				
BMI	29.0 (5.8)	31.6 (6.2)	25.3 (4.5)	27.3 (5.3)
Current smokers, %	13.8	16.0	12.8	13.6
Physical activity, MET-hrs/week	9.6 (11.1)	8.7 (15.3)	16.4 (20.1)	13.0 (14.1)
Diet quality score	66.8 (10.6)	61.7 (11.1)	51.8 (10.0)	54.4 (9.6)

[1]While information on diabetes, antihypertensive medications, and lipid-lowering medications was available in NHS, we were unable to include them as covariates in the analysis because among the small sample of Black participants, no participant with depression endorsed these variables

medRxiv preprint doi: https://doi.org/10.64898/2026.09.10.26362734; this version posted September 13, 2026. The copyright holder for this preprint (which was not certified by peer review) is the author/funder, who has granted medRxiv a license to display the preprint in perpetuity.
It is made available under a CC-BY 4.0 International license .

Supplemental Table 4. Metabolites nominally associated with depression status among MESA Black participants in either of the two multivariable models

Metabolites	Model 1			Model 2		
	Estimate	P-value	FDR-adjusted	Estimate	P-value	FDR-adjusted
Serotonin	-0.39132	2.67E-05	0.009952	-0.44871	4.33E-06	0.001777
Hydroxytryptophan	-0.39493	3.02E-06	0.002251	-0.39609	4.77E-06	0.001777
Adenosine	-0.28438	0.000727	0.180594	-0.31094	0.000363	0.090041
Trigonelline	0.251698	0.004115	0.594614	0.247234	0.006185	0.671408
Inosine	-0.25705	0.005861	0.594614	-0.25131	0.007154	0.671408
CAR 12:2	-0.23966	0.007176	0.594614	-0.2392	0.008826	0.671408
LPC 18:1/0:0	0.216973	0.016398	0.594614	0.258705	0.009394	0.671408
DG 34:0	0.247923	0.004208	0.594614	0.23692	0.010687	0.671408
LPE 18:0/0:0	0.221955	0.013527	0.594614	0.244603	0.01208	0.671408
DG 38:5	0.222991	0.009209	0.594614	0.237448	0.01278	0.671408
Tauromuricholic acid	0.246355	0.011316	0.594614	0.241818	0.013555	0.671408
CAR 14:3	-0.22573	0.009145	0.594614	-0.21627	0.014526	0.671408
Methionine sulfoxide	0.202377	0.019215	0.594614	0.212384	0.015756	0.671408
LPE 20:4/0:0	0.226886	0.010954	0.594614	0.231878	0.015853	0.671408
LPC 20:4/0:0	0.212107	0.019247	0.594614	0.241812	0.016082	0.671408
Arachidic acid	0.214932	0.013161	0.594614	0.217525	0.016393	0.671408
PC 34:1	0.198983	0.024727	0.594614	0.251572	0.016982	0.671408
TG 51:4	-0.19099	0.029306	0.594614	-0.22232	0.017268	0.671408
ATP	-0.24409	0.016663	0.594614	-0.24519	0.01744	0.671408
LPE 16:0/0:0	0.21189	0.018577	0.594614	0.22189	0.019	0.671408
CAR 14:2	-0.21479	0.015845	0.594614	-0.21285	0.019232	0.671408
N1-methyl-2-pyridone-5-carboxamide	0.192744	0.022464	0.594614	0.203996	0.020587	0.671408
CAR 16:3	-0.18616	0.034501	0.604869	-0.20875	0.020728	0.671408
S-Adenosyl-L-homocysteine	0.248916	0.017201	0.594614	0.245205	0.022552	0.700053
Nonadecylic acid	0.218122	0.018405	0.594614	0.215676	0.023842	0.71048
Caffeine	0.204807	0.027068	0.594614	0.212215	0.024939	0.714599
TG 40:0	0.21762	0.02038	0.594614	0.214839	0.027676	0.732322

medRxiv preprint doi: https://doi.org/10.64898/2026.09.10.26362734; this version posted September 13, 2026. The copyright holder for this preprint (which was not certified by peer review) is the author/funder, who has granted medRxiv a license to display the preprint in perpetuity.
It is made available under a CC-BY 4.0 International license .

LPE 18:1/0:0	0.203083	0.020882	0.594614	0.206441	0.02801	0.732322
CAR 12:3	-0.16339	0.039783	0.604869	-0.17951	0.028506	0.732322
DG 38:4	0.223018	0.010604	0.594614	0.203523	0.031215	0.760053
CAR 10:1	-0.19111	0.031073	0.604869	-0.19415	0.032232	0.760053
4-Acetamidobutanoic acid	0.199831	0.027292	0.594614	0.194644	0.034944	0.760053
N-Acetyl-D-tryptophan	0.161642	0.070774	0.719679	0.190684	0.036453	0.760053
CAR 10:0;OH	-0.20631	0.027413	0.594614	-0.19998	0.037112	0.760053
Thiamine	0.184371	0.039041	0.604869	0.19258	0.037357	0.760053
LPE 22:6/0:0	0.177373	0.066804	0.719679	0.208685	0.038423	0.760053
Undecanedioic acid	-0.20763	0.048677	0.638176	-0.22243	0.038492	0.760053
p-Hydroxyphenylacetic acid	0.192834	0.028823	0.594614	0.187919	0.038768	0.760053
N1-Methyl-4-pyridone-3-carboxamide	0.178403	0.0365	0.604869	0.183283	0.040097	0.764269
LPC 0:0/22:5	0.161822	0.074355	0.719679	0.200822	0.042528	0.764269
Linoleoyl ethanolamide	-0.17234	0.039049	0.604869	-0.16933	0.043254	0.764269
Xanthosine	-0.19855	0.029024	0.594614	-0.18561	0.043998	0.764269
LPC 22:6/0:0	0.149022	0.109718	0.824785	0.20133	0.0446	0.764269
CAR 16:2	-0.18317	0.039205	0.604869	-0.1803	0.047023	0.764269
Malonic acid	-0.19416	0.023983	0.594614	-0.17227	0.047861	0.764269
PC P-34:1	0.141828	0.118971	0.853541	0.24736	0.049582	0.764269
Tridecylic acid	0.196524	0.029531	0.594614	0.181328	0.05203	0.764269
2-Hydroxystearic acid	0.204678	0.025957	0.594614	0.182084	0.054137	0.764269
Uridine	-0.22099	0.010753	0.594614	-0.17592	0.054371	0.764269
DG 32:0	0.188083	0.031928	0.604869	0.18348	0.059948	0.81202
Hypoxanthine	-0.18839	0.047396	0.638176	-0.18088	0.063169	0.814661
LPE 0:0/20:4	0.189471	0.039179	0.604869	0.184683	0.063712	0.814661
Cyclicamp	-0.18723	0.045863	0.638176	-0.17544	0.065361	0.814661
LPE 20:3/0:0	0.19132	0.046671	0.638176	0.179479	0.069436	0.814661
CAR 18:2	-0.18622	0.034027	0.604869	-0.15998	0.076469	0.814661
Dehydroepiandrosterone sulfate	-0.18543	0.036842	0.604869	-0.15789	0.079804	0.814661
Taurodeoxycholic acid	0.170557	0.043733	0.638176	0.148871	0.082123	0.814661
PE 34:1	0.202418	0.02531	0.594614	0.162016	0.085588	0.814661
Salicyluric acid	0.172353	0.048918	0.638176	0.153779	0.091855	0.814661
Palmitic acid	0.188914	0.045706	0.638176	0.164251	0.093279	0.814661

medRxiv preprint doi: https://doi.org/10.64898/2026.09.10.26362734; this version posted September 13, 2026. The copyright holder for this preprint **(which was not certified by peer review)** is the author/funder, who has granted medRxiv a license to display the preprint in perpetuity.
It is made available under a CC-BY 4.0 International license .

LTB3	0.191687	0.033615	0.604869	0.156104	0.097825	0.828175
PE 36:4	0.191412	0.047084	0.638176	0.135128	0.174164	0.933045

Model 1: adjusted for age, sex, study site, education, income, employment status

Model 2: Model 1 + adjusted for BMI, smoking status, physical activity, diet quality, systolic blood pressure, HDL cholesterol, total cholesterol, diabetes status, antihypertensive medications, and lipid-lowering medications

medRxiv preprint doi: https://doi.org/10.64898/2026.09.10.26362734; this version posted September 13, 2026. The copyright holder for this preprint (which was not certified by peer review) is the author/funder, who has granted medRxiv a license to display the preprint in perpetuity.
It is made available under a CC-BY 4.0 International license .
